# *In silico* characterisation of the avocado *WAK/WAKL* gene family with a focus on genes involved in defence against *Phytophthora cinnamomi*

**DOI:** 10.3389/fpls.2024.1474781

**Published:** 2025-01-29

**Authors:** Aaron Harvey, Noëlani van den Berg, Velushka Swart

**Affiliations:** ^1^ Department of Biochemistry, Genetics and Microbiology, University of Pretoria, Pretoria, South Africa; ^2^ Hans Merensky Chair in Avocado Research, Forestry & Agricultural Biotechnology Institute (FABI), University of Pretoria, Pretoria, South Africa

**Keywords:** *Persea americana*, wall-associated kinase, wall-associated kinase-like, promoter analysis, expression analysis, protein modelling, protein-ligand binding

## Abstract

The avocado industry faces a significant threat from the hemibiotrophic oomycete pathogen *Phytophthora cinnamomi*. A variably expressed defence gene during an avocado infection trial was a *Wall-associated kinase* (*WAK*). WAK and WAK-Like (WAKL) proteins are known to bind to fragmented pectin (oligogalacturonides) produced during pathogen penetration, thereby activating downstream defence-related pathways. To better understand the *P. cinnamomi*-avocado defence interaction, this gene family was assessed using *in silico* methods. In this study, previously generated RNA-sequencing data were used to associate genes with the defence response, followed by promoter- and phylogenetic analysis of these genes/proteins. The predicted proteins from these genes were modelled with AlphaFold2, and structural similarity across different rootstocks, as well as their binding affinity for oligogalacturonides, were assessed. The analysis identified 14 *Persea americana* (*Pa)WAK*s and 62 *PaWAKL*s across the West-Indian (pure accession reference), Dusa^®^, Leola™ and R0.12 avocado rootstock genomes. These genes showed distribution across the West-Indian genome’s chromosomes, with MCScanX analyses predicting tandem duplications. *PaWAK/WAKL* expression profiles were compared, implicating five *PaWAK/WAKL*s in defence. Phylogenetic and promoter analyses were conducted to predict associated defence-related pathways, focusing on stress and phytohormone-responsive pathways. Structural differences and varying oligogalacturonide binding affinities of PaWAK/WAKLs were predicted across rootstocks. These defence-related genes could be incorporated into a molecular screening tool to improve the development of resistant avocado rootstocks.

## 1 Introduction

South Africa is one of the greatest producers of *Persea americana* (avocado) in the southern hemisphere however production is significantly threatened by the soil-borne oomycete *Phytophthora cinnamomi* (Hardham, 2005; Hardham and Blackman, 2018; Sibulali, 2020). Infection by this hemibiotroph can lead to Phytophthora root rot, a disease that may result in decreased fruit yield and potential tree death (Ramírez-Gil et al., 2017a). In the nursery stage of this crop production, *P. cinnamomi* (as part of a wilt complex) can cause between 24.5-63.8% incidence and mortality (Ramírez-Gil et al., 2017b). Avocado deploys a multi-faceted defence response that includes cell wall modifications, Reactive Oxygen Species (ROS) scavenging and detoxification, proteinase inhibitors and induction of defence-related genes (Van Den Berg et al., 2021). A *Wall-associated kinase* (WAK) was identified as one of the defence- and stress-related genes with the most variable expression between the susceptible R0.12 and partially-resistant Dusa^®^ avocado rootstocks following *P. cinnamomi* inoculation (Backer et al., 2022). The *WAK* and *WAK-Like* (*WAKL*) gene family has been associated with defence responses in a variety of plant species. Examples that illustrate the extensive defence capabilities of *WAK/WAKLs* in defence against bacterial, fungal, viral and oomycete pathogens include *WAK/WAKL*s in tomato against *Pseudomonas syringae*, rose against *Botrytis cinerea*, cotton against *Verticillium dahliae* and *Fusarium oxysporum*, rice against *Magnaporthe oryzae, Nicotiana benthamiana* against tomato yellow leaf curl virus, and potato against *Phytophthora infestans* (Delteil et al., 2016; Wang et al., 2020; Zhang et al., 2020b; Liu et al., 2021; Yu et al., 2022; Zhong et al., 2023). Some WAK/WAKLs have been shown to confer resistance to fungi with either hemibiotrophic or necrotrophic lifestyles, through various mechanisms including pathogen- or host-derived elicitor detection and cell wall restructuring (Stephens et al., 2022).

WAK proteins are transmembrane pectin receptors, part of the 15 receptor-like kinase (RLK) subfamilies and serve several functions within the plant cell. WAKs can bind native pectin thereby influencing cell expansion during developmental stages. They can also bind oligogalacturonides (OGs), which are fragmented pectin produced during abiotic stress like wounding and biotic stress like pathogen infection. The OGs act as damage-associated molecular patterns (DAMPs) to elicit a defence response (Kohorn and Kohorn, 2012; Kohorn, 2015).

With the availability of the avocado West-Indian pure-accession reference genome and the re-sequenced genomes of key rootstocks (Avocado Genome Consortium), there is an opportunity to identify and compare the *Persea americana* (*Pa)WAK/WAKLs* among rootstocks with varying levels of resistance to *P. cinnamomi*. Given the importance of this gene family in plant defence across multiple species, this study aimed to identify a subset of these genes that will be implicated in the successful defence response of avocado against *P. cinnamomi* and that differences in this gene family between rootstocks contribute to variations in defence. This is the first comprehensive *in silico* characterisation of the *PaWAK/WAKL* gene family in avocado, revealing 14 *PaWAK* and 62 *PaWAKL*s. Five candidates (*PaWAK4*, *PaWAK13*, *PaWAKL10, PaWAKL29*, and *PaWAKL31)* were involved in defence, showing differences between the rootstocks in terms of expression during infection, *cis*-acting regulatory elements, predicted 3D protein structures and OG binding affinity (BA). The analyses revealed differences across rootstocks, indicating that *PaWAK/WAKL*s play a crucial role in the varying efficiencies of defence responses at a multi-omic level.

## 2 Materials and methods

### 2.1 Putative *PaWAK/WAKL* identification

Wall-associated kinases (WAK) or Wall-associated kinase-like (WAKL) protein sequences were extracted from the NCBI database, including those of AtWAK/WAKLs (https://www.ncbi.nlm.nih.gov/website, accessed on 15/06/2023). A Clustal Omega alignment (Geneious Prime v2023.1.2 with default settings) was performed to generate a Hidden Markov Model (HMM) profile, which was used as a query for a local HMMER v3.3.2 (Finn et al., 2011) search against the avocado West-Indian pure accession reference, Dusa^®^, Leola™ and R0.12 predicted proteomes (Avocado Genome consortium, unpublished data). Results with E-values < 1x10^-5^ were retained for further classification.

### 2.2 PaWAK/WAKL classification

All protein sequences were extracted from the West-Indian genome for protein domain analysis. Each sequence had to contain either a WAK or GUB_WAK_bind (Wall-associated receptor kinase galacturonan binding), STKc_IPAK (Catalytic domain of the Serine/Threonine kinases, Interleukin-1 Receptor Associated Kinases and related STKs) or PKc-like (Protein Kinases, catalytic domain) domain predicted by the NCBI Conserved Domain Database (CDD) search (https://www.ncbi.nlm.nih.gov/Structure/bwrpsb/bwrpsb.cgi). Additionally, each sequence required a GUB_WAK_Bind and Protein kinase (Pkinase) domain predicted by InterProScan (specifically the Pfam database https://www.ebi.ac.uk/interpro/) to be considered a WAK/WAKL. Sequences not meeting these criteria were excluded. Subsequent WAK classifications required a WAK or GUB_WAK and STKc_IPAK or PKc-like domain, a signal peptide predicted through SignalIP-5.0 (https://services.healthtech.dtu.dk/services/SignalP-5.0/), a transmembrane domain predicted through THMM-2.0 (https://services.healthtech.dtu.dk/services/TMHMM-2.0/), and at least one EGF (epidermal growth factor)-like or EGF_CA (EGF-calcium binding) domain predicted through NCBI CDD, InterProScan (Pfam), and Simple Modular architecture Research Tool (SMART - http://smart.embl-heidelberg.de/). Domains had to be present in two of three predictions with an E-value < 0.01 to be considered true domains. Sequences not containing these five domains in tandem were classified as WAKLs (Zhang et al., 2021; Li et al., 2022). The PaWAK/WAKLs were renamed according to position on the chromosomes. The protein domains, gene structure and position on the West-Indian genome of the *PaWAK/WAKL* genes were visualised with TBtools v2.086 (Chen et al., 2020).

### 2.3 Protein sequence analyses

The molecular weight and isoelectric point of the proteins, along with their subcellular localisation and conserved motifs, were predicted using the online platforms GeneInfinity (http://www.geneinfinity.org), WoLF PSORT (https://wolfpsort.hgc.jp/), and MEME. (https://meme-suite.org). The protein sequences of all the PaWAK/WAKLs were subjected to an All-to-All Blastp search, followed by the use of MXScanX (in TBtools) to predict the tandemly duplicated gene pairs (Wang et al., 2012; Chen et al., 2020). This analysis was further refined by predicting the non-synonymous substitution rate (K_a_), synonymous substitution rate (K_s_), and K_a_/K_s_ ratio for the tandemly duplicated pairs using TBtools v2.086. These predictions were used to determine which evolutionary processes, either purifying or positive selection, are acting on these gene pairs (Chen et al., 2020).

### 2.4 Expression analysis during *P. cinnamomi* infection

RNA-sequencing data were used from a previous study, with NCBI GenBank repository accession number PRJNA675400 accessed on 15/06/2023 (Backer et al., 2022). In this study, avocado rootstocks were inoculated with a *P. cinnamomi* zoospore suspension, and roots were harvested at 6, 12, 24 and 120 hpi for RNA-sequencing. The expression profiles for all *PaWAK/WAKLs* at these four time points were obtained and visualised through a heatmap generated with R-studio and TBtools v2.086 (Racine, 2012; Chen et al., 2020).

### 2.5 Phylogenetic analysis

*Arabidopsis thaliana* WAK/WAKL protein sequences were obtained from The Arabidopsis Information Resource (TAIR) database (https://www.arabidopsis.org/, accessed on 11/07/2023) based on the designations previously described (Verica and He, 2002). AtWAKL19 was excluded as its sequence was not available on TAIR. Defence-related WAK and WAKL protein sequences were obtained from respective plant genome databases (**Supplementary Table S1**, accessed on 11/07/2023) based on relevant literature (Larkan et al., 2020; Wang et al., 2020; Zhang et al., 2020b; Liu et al., 2021; Stephens et al., 2022; Kong et al., 2023). These sequences were aligned with the PaWAK/WAKLs using Clustal Omega, and identical sites were masked. A Jukes-Cantor, Unweighted Pair-Group Method with Arithmetic Mean (UPGMA) phylogenetic tree was constructed using a bootstrap of 5000 (node threshold of 50%), with default settings on Geneious Prime v2023.1.2. The Newick tree was visualised with Interactive Tree of Life (iTOL v6, https://itol.embl.de/).

### 2.6 Promoter analysis

The promoter regions (2000 bp upstream of the start codon) of all identified *PaWAK* and *PaWAKL* genes were extracted from the West-Indian pure accession, Dusa^®^, Leola™, and R0.12 genomes using GenomeView-N42 (Abeel et al., 2012; Zhang et al., 2021; Li et al., 2022; Wang et al., 2023a; Zhong et al., 2023). These promoter sequences were uploaded to the PlantRegMap Binding site prediction tool (http://plantregmap.gao-lab.org/) with *A. thaliana* as the reference species and a P-value cutoff of < 10^-5^ (Lescot et al., 2002; Tian et al., 2019). Additionally, the promoter sequences were analysed using the PlantCARE online database (https://bioinformatics.psb.ugent.be/webtools/plantcare/html/). Identified elements with two or more predictions in the same location were considered as a single element. The identified elements were visualised with TBtools v2.086.

### 2.7 Structure and ligand binding predictions

The protein sequences corresponding to the upregulated genes were uploaded to the AlphaFold2 online platform for 3D structure prediction (https://colab.research.google.com/github/sokrypton/ColabFold/blob/main/AlphaFold2.ipynb#scrollTo=kOblAo-xetgx). AlphaFold2 was chosen because AlphaFold3 was still in Beta version at the time of article submission. The resulting protein structure PDB files were visualised with ChimeraX v1.7.1, and the protein domains were coloured according to the InterProScan Pfam protein domain predictions. The 3D protein structures of the same WAK/WAKL proteins across different genomes were superimposed using CLICK and TM-score server (http://cospi.iiserpune.ac.in/click/ and https://seq2fun.dcmb.med.umich.edu//TM-score/) to calculate superimposition scores between the pairs (Zhang and Skolnick, 2004; Nguyen et al., 2011). Through the CLICK analysis, lower to middling RMSD scores (< 2 Å to 2-4 Å) indicate high to moderate structural similarity, while higher to middling associated Z-scores (>2 and 1-2) suggest significant alignment better than random chance. A high RMSD score (>2 Å) with a low Z-score (<1) indicates structural differences and unreliable alignment. The RMSD scores follow the same boundaries in the TM-score sever analysis, with scores closer to one (cutoff boundary TM-score > 0.5), indicating that the proteins are more likely to be in the same fold (Xu and Zhang, 2010). To serve as negative and positive controls, different PaWAKLs from two genomes (PaWAKL17 from Dusa^®^ and PaWAKL61 from Leola™) and the same PaWAKL from the same genome (PaWAKL17 from Dusa^®^) were superimposed. The 3D protein structures of the PaWAK/WAKLs were uploaded to the Molecular Docking server platform (https://www.dockingserver.com/) along with an OG 3D structure (beta-D-Galactopyranuronic acid, pectin monomer from PubChem with accession number 441476: https://www.ncbi.nlm.nih.gov/) to predict binding energies and inhibition concentration (Ki) of the OG interaction with the PaWAK/WAKLs (Bikadi and Hazai, 2009; Harvey et al., 2024). Because this interaction does not involve an inhibitor as the ligand, Ki was interpreted as Kd (dissociation constant), reflecting the BA of the protein for the ligand. Free energy of binding quantifies the energy change or stability that occurs due to the binding taking place. Favourable ligand-protein binding is indicated as negative free energy of binding values, with the larger negative values (lower values) indicating greater favourability. The vdW + Hbond + desolv energy is a single value that quantifies the energy contributed by Van der Waals forces, hydrogen bonds, and desolvation energy, with larger negative values representing more stable/favourable interactions. The Kd represents the concentration at which half of the protein’s binding sites are occupied with the ligand, with lower Kd values suggesting tighter binding affinity and higher values representing weaker binding affinity. The target binding area on the PaWAK/WAKLs was centred at the GUB_WAK_bind domain. The target box started with a volume of 35 Å^3^ and was reduced to ensure the GUB_WAK_bind domain and minimal other amino acids were included. The best binding scenario was used for evaluation.

## 3 Results

### 3.1 The repertoire of *PaWAK/WAKLs*

**Figure 1:**
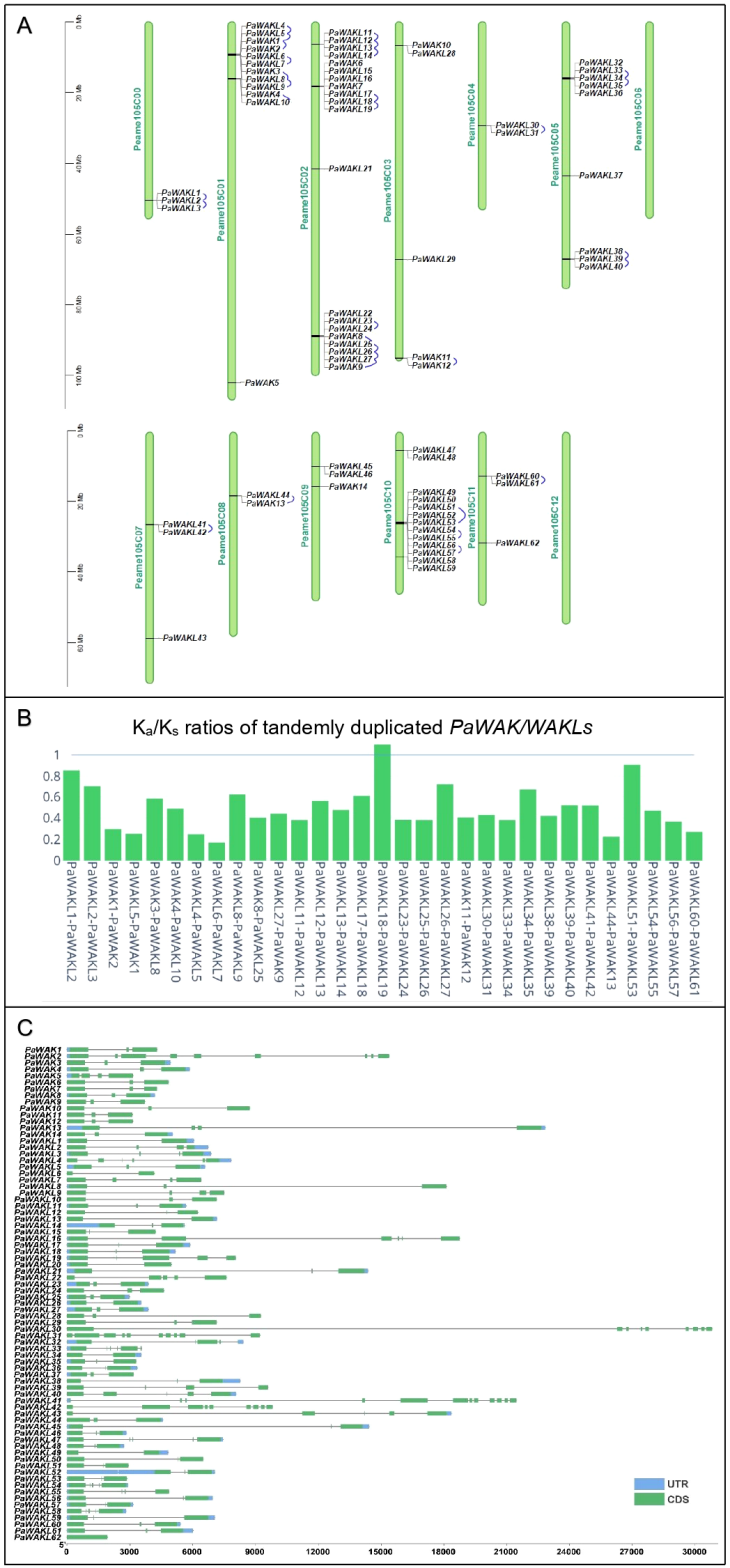
*PaWAK/WAKL* gene family characteristics. **(A)** The *PaWAK/WAKL*s are distributed across the 12 chromosomes and unassigned scaffolds (Peame105C00) of the West-Indian Pure accession reference genome, with tandemly duplication pairs connected by blue lines. **(B)** The K_a_/K_s_ ratio for the tandemly duplicated pairs was calculated using MCScanX. **(C)** The open reading frames of the *PaWAK/WAKL*s range from approximately 2000 to 30 000 bp, with varying numbers of exons (coding sequences - CDS) and introns (grey lines), and some untranslated regions (UTRs) identified. Visualisation with TBtools and Plotly (https://chart-studio.plotly.com).

The first step in the characterisation of the *PaWAK/WAKL*s is to identify, classify and describe the gene family members. The West-Indian reference genome contained 14 *PaWAKs* and 62 *PaWAKLs* that were randomly distributed, with some clustering across 10 of the 12 chromosomes and the unassigned scaffolds (**Figure 1A**), similar to observations in other species (Zhang et al., 2020a; Liu et al., 2021; Yu et al., 2022; Li et al., 2023; Yan et al., 2023; Harvey et al., 2024). There were 31 tandemly duplicated pairs predicted, with K_a_/K_s_ ratios between 0.17 and 1.10 (**Figure 1B**, **Supplementary Table S2**). One pair (*PaWAKL18* and *PaWAKL19*) had a ratio > 1, suggesting positive selection for this pair, unlike the rest of the gene pairs predicted to be undergoing purifying selection (K_a_/K_s_ < 1).

When comparing the genomes of R0.12, Leola™ and Dusa^®^ to the West-Indian reference, all 14 *PaWAKs* were identified in the other three genomes. However, the number of *PaWAKL*s varied: R0.12 had 60, Leola™ had 56 and Dusa^®^ had 58, with no additional *PaWAK/WAKLs* being identified (**Supplementary Table S3**). Dusa^®^ lacked *PaWAKL3, PaWAK6, PaWAK16*, and *PaWAK32;* R0.12 lacked *PaWAKL16* and *PaWAK39;* Leola™ lacked *PaWAKL12, PaWAK14, PaWAK15, PaWAK16, PaWAK21*, and *PaWAK53*. *PaWAKL16* is unique to the West-Indian genome while *PaWAKL39* is only absent in R0.12 (a *P. cinnamomi*-susceptible rootstock) but present in both partially-resistant rootstocks (Leola™ and Dusa^®^).

The *PaWAK/WAKL* open reading frames (ORFs) vary between 2,000 and 30,000 nucleotides with 1-10 exons and introns of differing lengths (**Figure 1C**). The gene architectures fall within the range of the previously described smallest (1.3 kb in *N. benthamiana*) and largest (~330 kb in *Juglans* species) *WAK/WAKLs* with exon numbers also showing similarities to other characterised *WAK/WAKLs*, such as the widest range of 1-27 exons in *Brassica rapa* (Zhang et al., 2020a; Li et al., 2022; Zhong et al., 2023; Harvey et al., 2024). The gene structure was well conserved across the *Arabidopsis thaliana* (*At*)*WAK/WAKL*s, with three exons and two introns, where the middle exon is smaller than the two flanking it (Verica and He, 2002). This gene structure pattern is seen in 11/14 *PaWAK*s and 29/62 *PaWAKL*s, representing the majority (40/62) of the gene structures observed. This structure, including those with four exons and three introns, was associated with immunity-related *WAK* genes in species like *A. thaliana*, cotton and rice (Stephens et al., 2022).

### 3.2 Protein properties and conserved motifs

**Figure 2:**
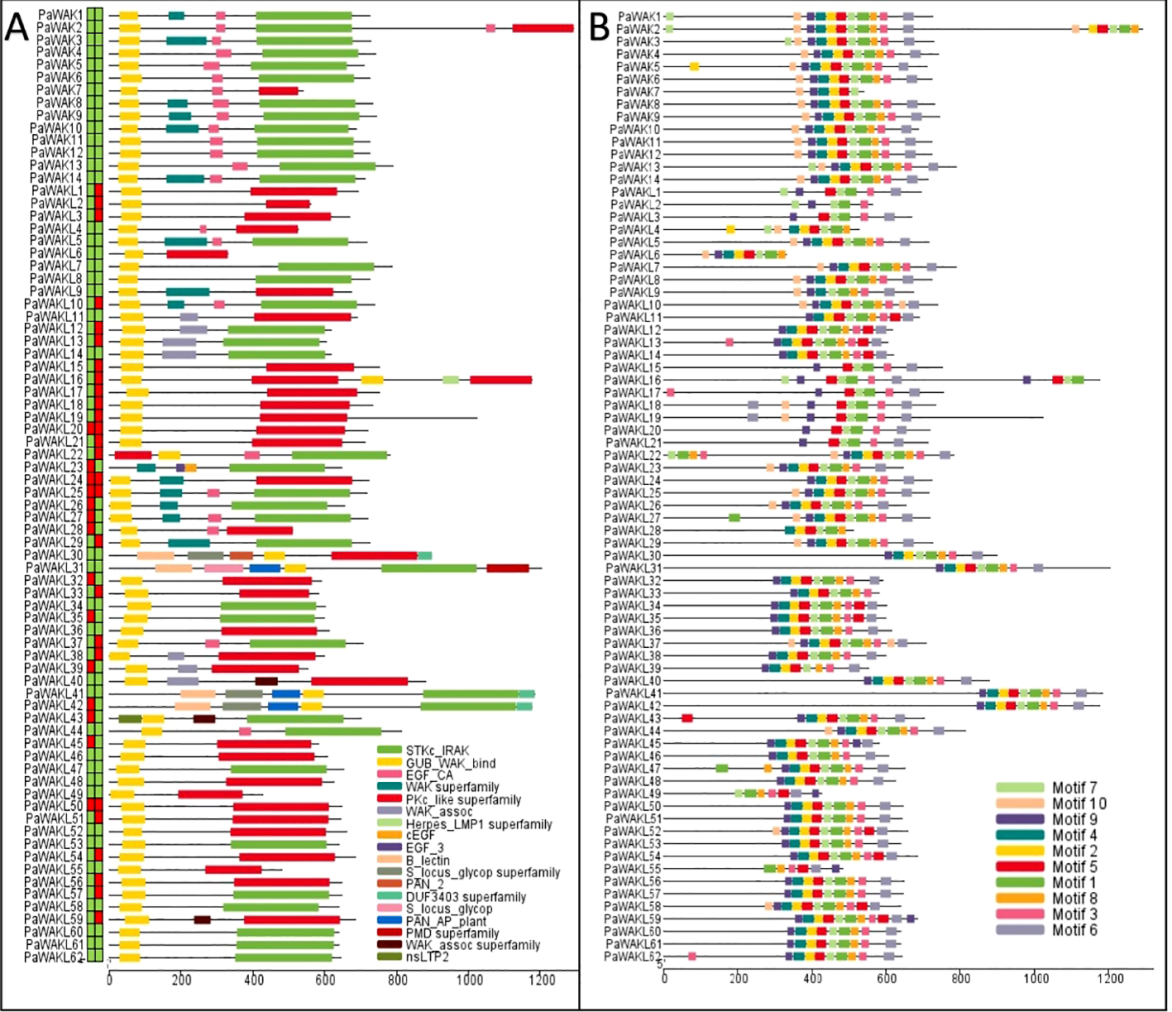
PaWAK/WAKL protein domains and conserved motifs. **(A)** PaWAK and PaWAKL protein domains were visualised using the results from the NCBI Conserved Domain Database (CDD) batch search. Signal peptides and transmembrane domains were predicted with the blocks preceding the proteins representing the presence (green) or absence (red) of each, respectively. **(B)** The top 10 most conserved protein motifs were predicted through MEME. Visualisations with TBtools.

The downstream PaWAK/WAKL proteins were also characterised to provide a multi-level description of this family. The proteins range from 301 to 1293 amino acids in length, have molecular weights between 33.45 and 142.86 kD, and isoelectric points between 5.17 and 9.13. The majority are predicted to localise to the plasma membrane, with some found throughout the cell (**Supplementary Figure S1**, **Supplementary Table S4**). These properties are within normal ranges for plant species, and plasma membrane localisation has been experimentally demonstrated in cotton and bread wheat WAK/WAKLs (Ramírez-Sánchez et al., 2016; Mohanta et al., 2019; Zhang et al., 2021; Xia et al., 2022). The PaWAK proteins contain the five required domains, while in the PaWAKLs multiple domain combinations are found, showing variation in the presence or absence of EGF_like, TM and SP domains. Specifically, 14/62 lack a signal peptide and 26/62 lack a transmembrane domain (**Figure 2A**, **Supplementary Table S3**).

PaWAKL5 was predicted to have an EGF_CA domain in the NCBI Conserved Domain Database (CDD) search with an E-value of ~0.002. However, this domain was not predicted through the Pfam database on InterProScan, and the SMART search E-value for the domain was 0.028 (E-value > 0.01, not considered true), thus it was classified as a WAKL. The 10 most abundant predicted conserved motifs in the PaWAK/WAKL proteins clustered within the C-terminal region, where the kinase domain lies (**Figure 2B**). When comparing the pairwise identity of the same WAK/WAKL protein sequences across the four genomes, identity varied from 10.5 to 100%, with more than half showing an identity above 80% (47/76). The protein sequence variance was mostly present in the PaWAKLs, while the PaWAK sequences were more conserved. This could suggest differences in the proteins produced by the same gene from different rootstocks.

### 3.3 Expression analysis during infection

**Figure 3:**
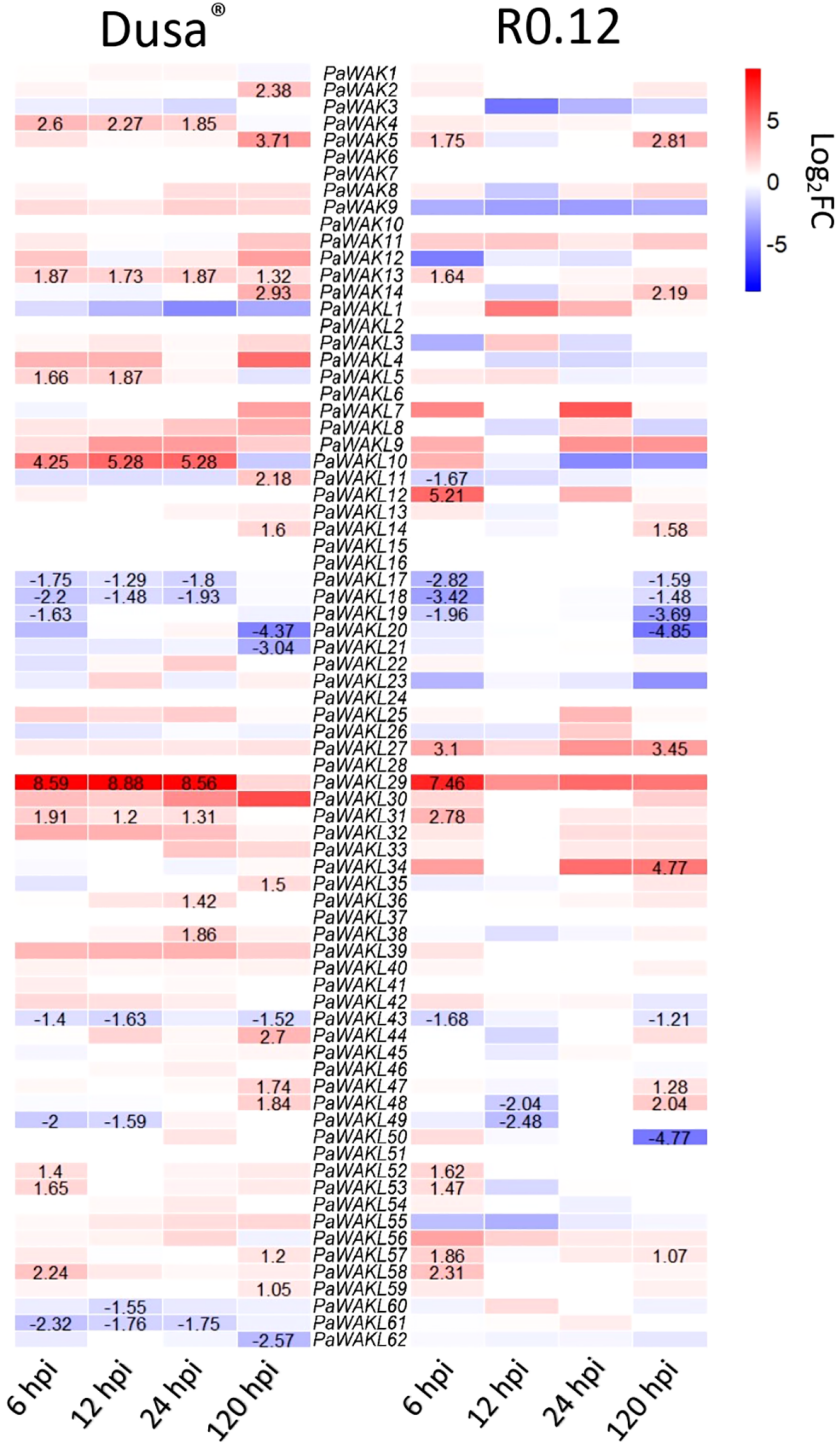
Expression profile heatmap of all *PaWAK/WAKL* genes during *Phytophthora cinnamomi* infection of a partially-resistant (Dusa^®^) and a susceptible (R0.12) avocado rootstock. More intense red blocks indicate a higher Log_2_FoldChange (Log_2_FC) and more intense blue blocks indicate a lower Log_2_FC compared to the controls. Values are shown for time points where the Log_2_FC change was significant, defined as P <0.05 and Log_2_FC > 1 or Log_2_FC < -1. All Log_2_FC values and P-values are provided in **Supplementary Table S5**.

The upregulation of certain genes during infection strongly implicates them in the defence response to pathogens. The *PaWAK/WAKL* expression profiles, from RNA-sequencing data (avocado inoculated with *P. cinnamomi*), showed differential expression within this gene family (**Figure 3**, **Supplementary Table S5**). Significant upregulation at one or more time points was observed for 22 (28.9%) *PaWAK/WAKLs* in Dusa^®^ and for 14 (18.4%) *PaWAK/WAKLs* in R0.12. In the partially-resistant avocado rootstock, Dusa^®^, *PaWAK4, PaWAK13, PaWAKL5, PaWAKL10, PaWAKL29*, and *PaWAKL31* were upregulated at multiple time points. Notably, *PaWAK13* and *PaWAKL29* also showed upregulation at 6 hours post-inoculation (hpi) in the susceptible R0.12. *PaWAKL29* exhibited a Log_2_Fold Change (Log_2_FC) > 8 at three time points (6, 12, 24 hpi) in Dusa^®^. In contrast, in R0.12, *PaWAKL29* expression was > 7 Log_2_FC at 6 hpi but then reduced to a non-significant increase compared to the experimental control. Significant downregulation at one or more time points was seen for 10 (13.2%) *PaWAK/WAKLs* in Dusa^®^ and for nine (11.8%) *PaWAK/WAKLs* in R0.12. Downregulation across two or more time points in Dusa^®^ was observed for *PaWAKL17, PaWAKL18*, *PaWAKL43, PaWAKL49*, and *PaWAKL61.* Of these, only *PaWAKL61* did not show downregulation in R0.12 as well.

### 3.4 Phylogenetic analysis

**Figure 4:**
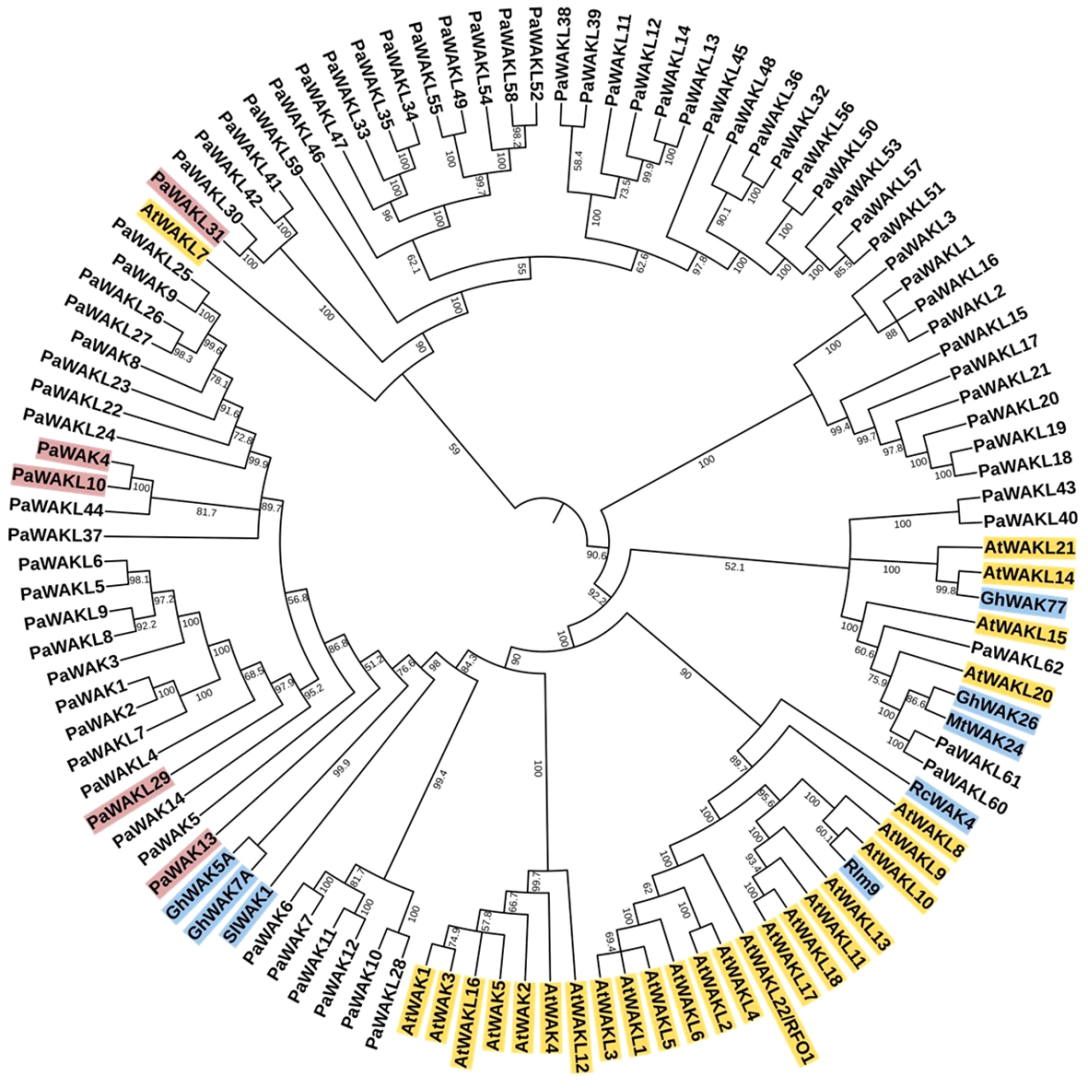
Phylogenetic analysis of the PaWAK/WAKL protein sequences with WAK/WAKLs from *Arabidopsis thaliana* and a subset associated with defence in rose, cotton, tomato, and barrel medic. This represents a Jukes-Cantor, Unweighted Pair-Group Method with Arithmetic Averaging (UPGMA) phylogenetic analysis of PaWAK/WAKL, AtWAK/WAKL (yellow) protein sequences along with those implicated in defence from other species in blue (Larkan et al., 2020; Zhang et al., 2020b; Liu et al., 2021; Stephens et al., 2022; Kong et al., 2023). Bootstrap support values are shown preceding the nodes (rounded to one decimal place). Protein sequences highlighted in red correspond to genes that showed upregulation during *Phytophthora cinnamomi* infection.

The Jukes-Cantor, Unweighted Pair-Group Method with Arithmetic Averaging (UPGMA) phylogenetic tree was constructed to determine if any PaWAK/WAKL shows a relationship to a previously characterised proteins allowing for functional inferences (**Supplementary Table S1**). The analysis revealed that the majority of the WAK/WAKLs from other species clustered separately from the PaWAK/WAKLs (**Figure 4**). Most of the AtWAK/WAKLs formed distinct clusters, some of which included WAK/WAKLs from other species. However, AtWAKL7, AtWAKL14, AtWAKL15, AtWAKL20, AtWAKL 21, and most of the defence-related WAK/WAKLs included in the analysis (excluding Rlm9 and RcWAK4) were dispersed within the PaWAK/WAKL clusters.

The protein sequences corresponding to the *PaWAKs* and *PaWAKLs* genes that are located near each other on the genome are grouped together in the phylogenetic analysis. This suggests that sequences in close genomic proximity tend to be more similar than those further apart. This correlates with the high number of tandemly duplicated gene pairs, as this expansion allows for duplication near the original sequence. An example is the clustering of *PaWAK9* and *PaWAKL25*, *PaWAKL26*, and *PaWAKL 27*, which are all located in proximity on the chromosome, with *PaWAKL25* and *PaWAKL26*, and *PaWAKL26* and *PaWAKL27* identified as tandemly duplicated gene pairs. The phylogenetic analysis supports the evidence of tandem duplications within this cluster. The node bootstrap confidence values range from 51.2 to 100, with the majority being over 70 (88/102 nodes > 70), allowing for functional inferences – sequences closely related may have similar functions within the plant cell. The closest relationship between the PaWAK/WAKL proteins resulting from the upregulated genes and a defence-related protein from another species is between PaWAK13 with *Gossypium hirsutum* (cotton) GhWAK5A, GhWAK7A and a *Solanum lycopersicum* (tomato) SlWAK1 (Wang et al., 2020; Zhang et al., 2020b). Pairwise comparisons of the protein sequence of PaWAK13 with SlWAK1, GhWAK5A and GhWAK7A, showed percent identities of 42.2, 44.8 and 42.2%, respectively, which is relatively low. The moderate relationship with low percent identities suggests that, while these protein sequences are the closest related within this subset, they may not share the same functions, as they differ by more than half of their sequence. With the limited relationships between the upregulated PaWAK/WAKLs proteins and the defence-related sequences from the other species, the functional inference here is minimal.

### 3.5 *Cis*-acting elements predictions

**Figure 5:**
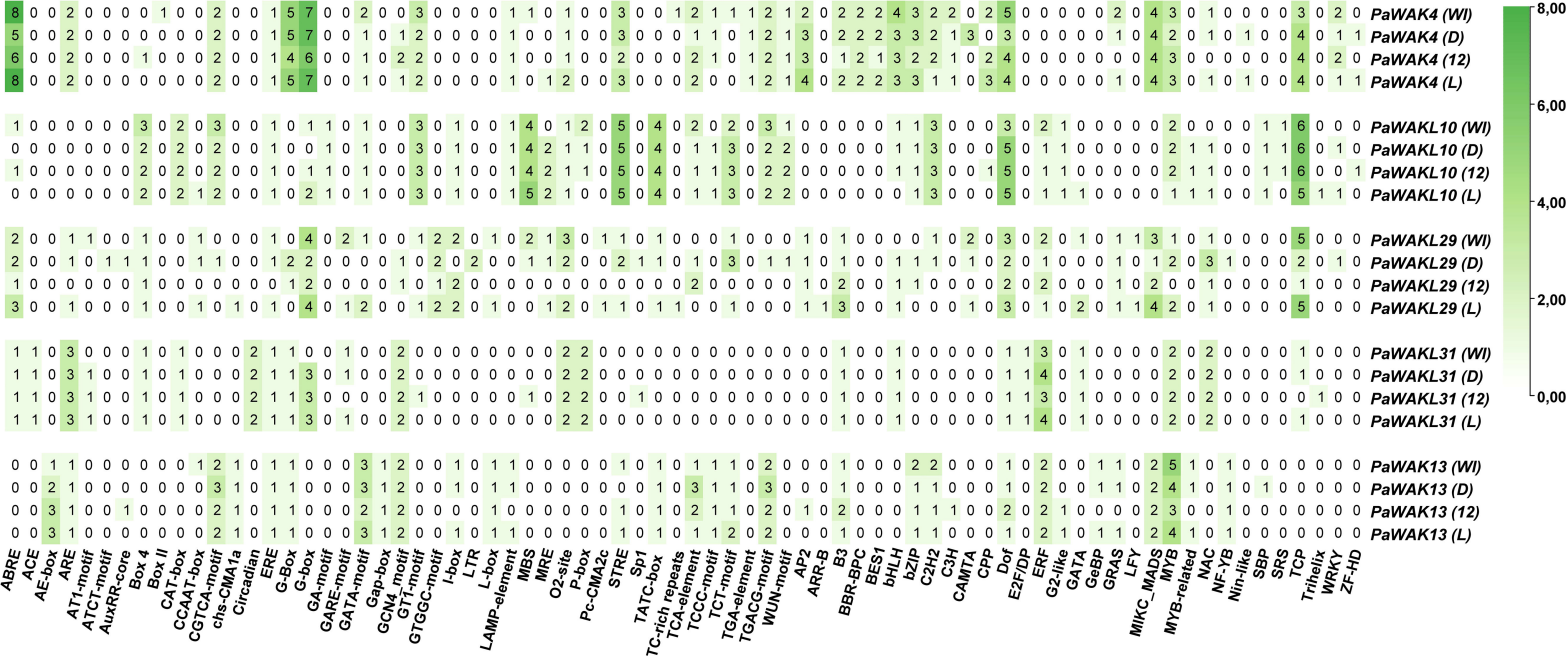
*Cis-*acting element composition in the promoter regions of *PaWAK4, PaWAKL10, PaWAKL29, PaWAKL31*, *and PaWAK13* across four avocado rootstock genomes (WI-West Indian, D-Dusa^®^, 12-R0.12 and L-Leola™) as predicted by PlantCARE (ABRE to TGACG-element) and PlantRegMap (AP2 to ZF-HD). More intense green indicates a greater number of unique locations for that particular element, with the value representing the number of unique locations predicted.

To understand the factors influencing the differential expression between Dusa^®^ and R0.12 during infection, the promoter regions (2000 bp upstream of the start codon) of the upregulated genes (*PaWAK4*, *PaWAK13*, *PaWAKL10*, *PaWAKL29*, and *PaWAKL31*) were analysed for the presence and composition of *cis*-acting elements (**Figure 5**). The minimal elements in the promoter of *PaWAKL29* in R0.12 are due to a large portion of the promoter consisting of unannotated nucleotides (N), making it difficult to draw conclusions about *PaWAKL29* regulation in R0.12. While the focus was the elements identified in the upregulated gene’s promoters, the full repertoire for all *PaWAK/WAKLs* was predicted (**Supplementary Table S6**). In total, 74 unique elements were identified with roles in light- (23 elements), phytohormone- (10 elements), stress-responsiveness (three elements), general processes (five elements) and floral development (three elements). Certain elements such as ACE, AE-box, ATCT-motif, AuxRR-core, Box II, Circadian, GA-motif, Gap-box, GTGGC-motif, LTR, Pc-CMA2c TGA-element, ARR-B, BBR-BPC, BES1, EF2/DP, GeBP, LYF, Nin-like, and SRS showed presence in only one gene (gene specificity) when considering the five upregulated gene promoter regions. Elements found in high abundance (three or more) in the *PaWAK4* promoter regions across the genomes include ABRE, G-Box, G-box, STRE, AP2, bHLH, bZIP, Dof, MICK_MADS, MYB and TCP. These elements are involved in phytohormone responsiveness, growth/development, and environmental responses (Tian et al., 2019). Notably, ABRE, an abscisic acid-responsive element, is more abundant in *PaWAK4* than in the other four genes, potentially indicating a pathway that differentiates its expression from the others. *PaWAKL10* has fewer elements compared to *PaWAK4*, with the same high abundance of Dof and TCP. However, *PaWAK4* lacked the TATC-box (involved in gibberellin-responsiveness), which *PaWAKL10* contained in four copies, conserved across the promoters from the four genomes. *PaWAKL29, PaWAKL31* and *PaWAK13* had fewer elements overall in this subset of promoters. When comparing Dusa^®^ promoters to R0.12, there are 11 elements in higher number in *PaWAK4*, one in *PaWAKL10*, 25 in *PaWAKL29*, three in *PaWAKL31* and 11 in *PaWAK13*. Alternatively, there are eight, four, five, four and six, respectively, in higher abundance in R0.12 promoters as compared to Dusa^®^. This illustrates the differences in promoter composition between these two rootstocks, which could be contributing to different levels of involvement in different cellular pathways.

The promoter regions of an additional partially-resistant avocado rootstock, Leola™, were included to predict the pathways involved in the same upregulated genes as Dusa^®^, revealing differences in composition compared to the other three rootstocks. The Leola™ *PaWAK4* promoter contained additional ABRE and STRE elements compared to R0.12, and additional ABRE elements compared to Dusa^®^, indicating a higher responsiveness to abscisic acid (ABA) and stress-related pathways. The Leola™ *PaWAKL10* promoter had a CCAAT-box (stress-responsive element) that was absent in all other promoters. Similar to the Dusa^®^ *PaWAKL31* promoter, the Leola™ promoter had a GARE-motif (Gibberellin responsive element), which was absent in R0.12. The *PaWAK13* promoter in Leola™ did not contain any phytohormone- or stress-responsive elements in higher abundance compared to R0.12, but it did have more GATA-motif, I-box, Lamp-element, and TCT-motif elements, which are all light-responsive. This suggests that *PaWAK13* in Leola™ may be more responsive to changes in light, potentially related to circadian pathways.

### 3.6 Structure and ligand binding analyses

**Figure 6:**
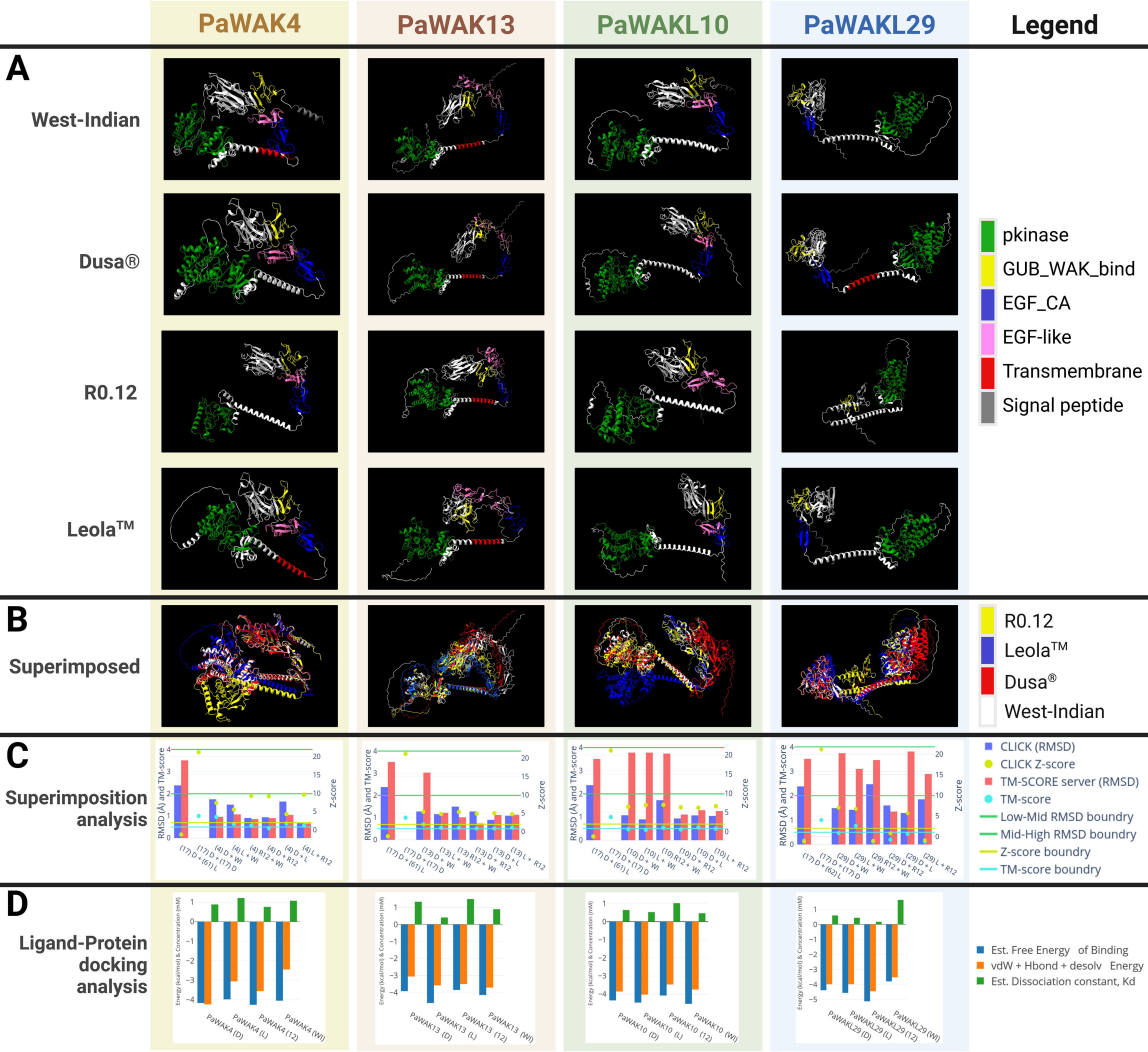
Comparison of the protein structures of PaWAK4, PaWAK13, PaWAKL10, and PaWAKL29 across four rootstocks, and their affinity to bind oligogalacturonides. **(A)** Visualisation of the 3D structure of PaWAK4, PaWAK13, PaWAKL10 and PaWAKL29 **(B)** The visual superimposition of the four predicted protein structures from four different rootstock genomes. **(C)** Root mean square deviation (RMSD) scores with associated Z-scores and TM-Scores to assess the similarity of PaWAK/WAKL proteins. Boundary lines for RMSD values (green line at 2 Å and 4 Å), Z-score (yellow line at 2 Å), and TM-score (light blue line at 0.5 Å) indicate critical thresholds. Gene names are summarised as (17) for PaWAKL17, (4) for PaWAK4, (13) for PaWAK13, (18) for PaWAKL18 and (29) for PaWAKL29 in the x-axis. Different PaWAKLs from two genomes ‘(17) D + (61) L’ and the same PaWAKL from the same genome ‘(17) D + (17) D’ were included to act as a negative and positive control. **(D)** Predicted ligand-protein binding of PaWAK/WAKLs and a pectin monomer (oligogalacturonide, PubChem accession: 441476). Energies involved with the precited binding are shown in blue and orange as negative values while the estimated dissociation concentration (Kd) is shown in green as positive values. Rootstocks: WI-West Indian, D-Dusa^®^, 12-R0.12 and L-Leola™. Graphs visualised with Plotly (https://chart-studio.plotly.com), 3D models visualised with ChimeraX and images created with BioRender.com.

The 3D protein structures of PaWAK4, PaWAK13, PaWAKL10, and PaWAKL29 from the four available rootstocks were visualised to assess structural similarity. PaWAKL31 was excluded from this analysis as this protein from multiple rootstocks could not be modelled with AlphaFold2. Visual representation of these protein structures revealed a lack of structural consistency across the rootstocks of the same PaWAK/WAKL proteins (**Figure 6A**). Amongst the proteins analysed, PaWAK13 showed the most similarity, while PaWAKL29 was the least similar. When superimposed, the same PaWAK or PaWAKL proteins from all four rootstocks demonstrated even more evident structural discrepancies (**Figure 6B**). Complete superimposition was not achieved for any of the four PaWAK/WAKLs across the rootstocks, only highlighting qualitative structural differences.

Pairs of 3D protein structures from two rootstocks at a time (an example would be R0.12 PaWAK13 superimposed with West-Indian PaWAK13) were assessed to determine if the proteins were structurally identical across the four rootstocks (**Figure 6C**, **Supplementary Table S7**). Most pairs of superimposed PaWAK4, PaWAK13 and PaWAKL10 had root mean square deviation (RMSD) values < 2 Å (Armstrong) with associated Z-scores > 2 and TM-scores > 0.5, indicating high structural similarities between these proteins across the rootstocks. However, PaWAKL29 proteins showed the most variation with only the PaWAKL29 in Leola™ and West Indian predicted to be structurally similar. The comparisons of PaWAK4 in Dusa^®^ and R0.12, PaWAK10 in Dusa^®^ and R0.12 and PaWAK4 in Leola™ and R0.12 showed TM-scores < 0.5, but all other values indicated structural similarity. The comparisons of PaWAK13 in Dusa^®^ and West Indian, PaWAK10 in R0.12 and West Indian, and PaWAKL29 in Leola™ and West Indian comparisons had RMSD values > 2 Å from the TM-score server, although the CLICK server RMSD scores were < 2 Å with high Z-scores and TM-scores, indicating structural similarity. The comparisons of PaWAKL10 in Leola™ and West Indian, PaWAKL29 in Dusa^®^ and West Indian and PaWAKL29 in Dusa^®^ and Leola™ had RMSD values > 2 Å from the TM-score server, with associated low TM-scores, but a low RMSD from CLICK (closer to 2 Å than any other PaWAKL10 comparison), and high Z-scores. The low Z- and TM-scores for PaWAKL29 in R0.12 and West Indian, PaWAKL29 in Dusa^®^ and R0.12 and PaWAKL29 in Leola™ and R0.12 suggest that these pairs consist of structurally different proteins. This provides quantitative evidence to suggest that the same protein can differ structurally in different rootstocks, which can lead to downstream effects.

A PaWAK/WAKL-OG binding prediction analysis was conducted to assess the binding potential of each corresponding upregulated gene’s PaWAK/WAKL protein with an OG and to identify if any proteins show higher affinity for the OG ligand, given the structural differences between rootstocks. All the PaWAK/WKALs assessed had negative binding energies between -4 and -5 kcal/mol, indicating favourable exothermic (spontaneous) binding (**Figure 6D**, **Supplementary Table S8**). There are binding energy and dissociation constant (Kd) differences between the same PaWAK/WAKL proteins from different rootstocks, likely due to the structural differences noted previously. Proteins that efficiently bind OGs will have larger negative free energy and vdW+Hbond+desolv energy, along with a smaller positive Kd value. The proteins predicted to bind OGs most efficiently from each rootstock are PaWAK4 in R0.12, PaWAK13 in Leola™, PaWAKL10 in West-Indian and PaWAKL29 in R0.12. However, the Dusa^®^ and Leola™ versions of PaWAK13 and PaWAKL10 are predicted to bind OGs more efficiently than their R0.12 equivalents.

## 4 Discussion

This study aimed to characterise the *PaWAK/WAKL* gene family and investigate a subset of its members involved in the successful defence response against *P. cinnamomi*. Fourteen *PaWAKs* and 62 *PaWAKLs* were identified and characterised across four avocado genomes. Among these, *PaWAK4, PaWAK13*, *PaWAKL10, PaWAKL29*, and *PaWAKL31* showed significant upregulation in the partially-resistant rootstock, Dusa^®^, at three or four time points during infection. The protein products of these candidate genes displayed variation in 3D structure and predicted ability to bind OGs across the rootstocks. Additionally, the two partially-resistant rootstocks, Dusa^®^ and Leola™, exhibited predicted differences, suggesting that their defence responses differ.

### 4.1 *In silico* characterisation of the *PaWAK/WAKL* gene family

The repertoire of 14 *PaWAK* and 62 *PaWAKLs* identified in avocado is similar to that identified in *Brassica rapa* but represents more than *Arabidopsis* and fewer *WAK*s than apple and pea (Verica and He, 2002; Zuo et al., 2019; Zhang et al., 2020a; Li et al., 2023). The classification definitions used in this study, based on protein domains, are not uniform across recent literature but represent an amalgamation to ensure a comprehensive protocol as described in the materials and methods section and in a recent review (Zuo et al., 2019; Zhang et al., 2020a; Liu et al., 2021; Li et al., 2022; Sipahi et al., 2022; Xia et al., 2022; Yu et al., 2022; Kong et al., 2023; Li et al., 2023; Wang et al., 2023a; Yan et al., 2023; Zhong et al., 2023; Harvey et al., 2024). *PaWAKL5* is an outlier to this protocol – according to an NCBI CDD search, it would be classified as a WAK, but according to the Pfam and SMART database, it would be classified as a WAKL. For this study, PaWAKL5 was classified as a WAKL, but this classification can be re-evaluated in future as protein domain databases and their predictions improve. There were differences in gene family composition across the four avocado genomes, with four, two and seven *PaWAKLs* not identified in Dusa^®^, R0.12 and Leola™, respectively (**Supplementary Table S3**). Molecular validation of these discrepancies should be done in the future to determine if they are true differences or due to annotation errors.

This clustering of genes (within its gene family) on the chromosomes can indicate tandem duplication which produces identical sequences adjacent to the original sequence. These events allow for the expansion of this gene family within species (Kong et al., 2007; Lallemand et al., 2020). Tandemly duplicated pairs have been predicted in bread wheat, walnut, cotton, cannabis, and potato (Dou et al., 2021; Zhang et al., 2021; Li et al., 2022; Sipahi et al., 2022; Xia et al., 2022; Yu et al., 2022). In avocado, the only duplicated pair predicted to be under positive selection was *PaWAKL18* and *PaWAKL19*. Interestingly, *PaWAKL18* is also a tandemly duplicated pair with *PaWAKL17*, but this pair is predicted to be under purifying selection (**Figure 1B**). A hypothesis for the positive selection of *PaWAKL18* is that it allows for neo-functionalization, where a new or different function arises for the duplicated sequence. In contrast, purifying selection allows for sub-functionalization, the division of the parent gene’s function. Thus, *PaWAKL19* could have been tandemly duplicated from *PaWAKL18* and is undergoing selection to develop a novel function (Roulin et al., 2013). The phylogenetic analysis showed that most of the tandemly duplicated pairs grouped together, providing further evidence of their close relationships (**Figure 4**). The formation of mostly species-specific clades in the phylogenetic analysis indicates that this gene family has expanded independently in the different plant species, similar to observations in cotton, barley and tomato (Sun et al., 2020; Tripathi et al., 2021; Zhang et al., 2021).

The most abundant predicted subcellular localisation for the PaWAK/WAKLs was the plasma membrane, with 24 proteins (31.2%) localised there (**Supplementary Figure S1**). However, the remaining proteins were localised across seven other cellular locations, similar to predictions for the *Cannabis sativa* WAK/WAKLs (Sipahi et al., 2022). Although all PaWAKs contained a TM domain and signal peptide, not all of them were predicted to be localised at the plasma membrane. This suggests that the PaWAK/WAKLs perform their functions throughout the cell and are not restricted to the plasma membrane, potentially embedding in other membranes. This could allow PaWAK/WAKLs to detect OGs after cell wall damage, where OGs could enter the cell along with the pathogen. PaWAK/WAKLs embedded in other organelle membranes could activate pathways specific to those organelles. For example, PaWAK/WAKLs localising to the mitochondria could influence ROS production, a downstream effect proposed for GhWAK7A-mediated chitin signalling against fungal pathogens in cotton (Wang et al., 2020). Another potential explanation is that the PaWAK/WAKLs are recognising the trafficking of pathogen proteins in the cytoplasm, interacting with the membrane of vesicles that effectors have used to enter the cell through endocytosis pathways (Wang et al., 2023b). The conserved motifs were mostly localised to the C-terminal of the PaWAK/WAKLs, within the kinase domain, suggesting that the kinase domain has the largest conservation across the proteins, similar to observations in barley, cannabis, cotton, pea, potato, sesame, and tomato (Kurt et al., 2020; Tripathi et al., 2021; Zhang et al., 2021; Sipahi et al., 2022; Yu et al., 2022; Li et al., 2023; Yan et al., 2023). The kinase domain performs the fundamental function of phosphorylating proteins, which could explain the minimal variation in these regions. In contrast, the N-terminal showed more variation and contained the GWB and EGF domains. This variation in the N-terminal might allow PaWAK/WAKLs to have specialised cellular roles, enabling a more targeted response to environmental or developmental changes.

### 4.2 *PaWAK/WAKL*s implicated in avocado defence against *Phytophthora cinnamomi*

There were 31 differentially expressed *PaWAK/WAKLs* in Dusa^®^ and 23 in R0.12 at least at one timepoint, with a total of 35 unique genes indicating that this family is responsive to *P. cinnamomi* infection (**Figure 3**). *PaWAK4*, *PaWAK13*, *PaWAKL10*, *PaWAKL29*, and *PaWAKL31* showed significant upregulation across three or four time points in the partially-resistant rootstock Dusa^®^. In contrast, the susceptible R0.12 showed upregulation of *PaWAK13*, *PaWAKL29*, an*d PaWAKL 31* only at 6 hpi. These genes are not clustered together in the phylogenetic analysis, except for *PaWAK4* and *PaWAKL10*, which show a very close relationship and were predicted to be a tandemly duplicated pair with a K_a_/K_s_ ratio < 1 (**Figures 1**, **4**). It is possible that *PaWAKL10* was tandemly duplicated from *PaWAK4* and has now undergone sub-functionalization, with both acting together during *P. cinnamomi* infection. The main difference in expression patterns between the two rootstocks is the duration of differential expression. All *PaWAK/WAKLs* showing significant up- or down-regulation at 6 hpi in R0.12 subsequently returned to non-significant differential expression levels by 12 hpi. In contrast, most differentially expressed *PaWAK/WAKLs* in Dusa^®^ at 6 hpi maintained their significant up- or down-regulation across multiple time points. This prolonged differential expression in Dusa^®^ could contribute to its successful defence response compared to R0.12.

A phylogenetic analysis was conducted using the PaWAK/WAKLs, AtWAK/WAKLs and dicotyledonous plant WAK/WAKLs, previously implicated in defence, to identify relationships between the proteins and to infer function (**Figure 4**). Most defence-related sequences clustered with the AtWAK/WAKLs, showing minimal dispersion with the PaWAK/WAKLs. Notably, PaWAKL40, PaWAKL 43, PaWAKL 60, PaWAKL 61, and PaWAKL 62 are clustered with two cotton GhWAKs, four AtWAKLs and a *Medicago truncatula* (Mt)WAK. However, all corresponding genes, except those for PaWAKL40, were significantly downregulated, at least at one timepoint in Dusa^®^ suggesting they do not contribute to a successful defence response. PaWAK13 exhibited a moderate relationship with cotton GhWAK5A, GhWAK7A and a tomato SlWAK1. GhWAK7A is involved in cotton defence against two fungal pathogens, a hemibiotroph (*Verticillium dahlia*) and a nectrotroph (*Fusarium oxysporum*), through chitin signalling pathways. GhWAK5A showed upregulation seven days post *V. dahliae* inoculation, although the downstream pathway remains unknown (Wang et al., 2020). Despite the low protein percent identity and structural similarity between PaWAK13, GhWAK5A and GhWAK7A (44.87 and 42.2%, respectively, with RMSD values > 3.6 and TM-score < 0.18), it is the closest phylogenetic relationship in this subset and so there could be functional similarities as both are also associated in a hemibiotrophic pathogen defence response.

The *cis*-acting element composition in the promoter regions of upregulated genes was assessed to predict which pathways contribute to the observed differential expression and to compare them (**Figure 5**). Although expression data for the *PaWAK/WAKL*s in Leola™ are unavailable, the *cis*-acting elements can still provide evidence suggesting which pathways are involved in their regulation. Salicylic- (SA), jasmonic- (JA), ABA, and auxin (phytohormones) were previously linked to the successful defence response of avocado towards *P. cinnamomi* (Van Den Berg et al., 2018). It would be informative to determine if the *PaWAK/WAKLs* have elements in their promoter regions responsive to these phytohormones to infer which pathways are involved in their expression. The increased number of TCA-elements (SA-responsive element) in the Dusa^®^ *PaWAK13* promoter compared to the R0.12 promoter could partly explain the differential expression observed between these two rootstocks. The CGTCA- and TGACG-motifs (the positive and negative promoter strand), are responsive to methyl jasmonate (MeJA) and are more abundant in the Dusa^®^ *PaWAK13* promoter compared to the R0.12 promoter. This suggests that the Dusa^®^ *PaWAK13* promoter is more responsive to MeJA or JA than the R0.12 promoter (**Figure 5**). The ABA-responsive element, ABRE, is more abundant in *PaWAK4* compared to other upregulated gene promoters, indicating that it is more responsive to ABA than the other genes, with the Leola™ promoter having the most elements, suggesting it could be the most responsive to ABA. However, ABRE is more numerous in R0.12 compared to Dusa^®^, suggesting this pathway may not be responsible for the increased expression of *PaWAK4* in Dusa^®^ during *P. cinnamomi* infection.

Another important set of *cis*-acting elements to consider are the stress-responsive elements, namely CCAAT-box, STRE and TC-rich repeats. The TC-rich repeat elements are minimally present in promoter regions of the upregulated genes, but the Leola™ *PaWAKL29* promoter has them, unlike the R0.12 and Dusa^®^ promoters. The CCAAT-box is present in Leloa™ *PaWAKL10* and *PaWAKL29* and absent in the promoters of these genes in R0.12, suggesting that they are more stress responsiveness in Leola™ compared to R0.12 (and Dusa^®^), potentially contributing to its susceptibility. STRE is more abundant in the Dusa^®^ and Leola™ *PaWAK4* promoters compared to R0.12, indicating they may be more stress-responsive in the partially-resistant rootstocks. This could contribute to the differential expression observed in Dusa^®^ during the biotic stress response against *P. cinnamomi* infection.

### 4.3 Candidate defence PaWAK/WAKL analyses show structural and ligand-binding differences

The differences in expression and promoter *cis-*acting elements may not be the only factors contributing to varying defence efficiencies between rootstocks. Protein differences could also play a significant role. To explore this, the protein sequences of PaWAK4, PaWAK13, PaWAKL10, and PaWAKL29 were assessed to predict differences in protein structure and their ability to bind OGs. It is known that *P. cinnamomi* contains polygalacturonase (*PG*) genes, with its proteins involved in pectin cleavage, and three *PG*s show upregulation during infection of avocado, as observed in the RNA-sequencing data used in this study (Backer et al., 2022; Miyambo et al., 2022). This suggests that OGs are produced during infection, making it crucial to identify the proteins responsible for recognising them.

When visualised, these proteins across the rootstocks showed notable discrepancies between the rootstocks (**Figures 6A-C**). The comparison of PaWAK4 in Dusa^®^ and R12, PaWAKL10 in Dusa^®^ and R0.12 and PaWAKL10 in Leola™ and R0.12 showed TM-scores < 0.5. This suggests that these proteins have high structural similarity (more than expected by chance due to RSMD score) but may not lie in the same fold, as their 2D structures (α-helices and β-sheets) are in different spatial orientations. PaWAKL10 in the Leola™ and West Indian comparison highlights a contradiction that may arise from the prediction methods used. The Z-score, which measures the statistical significance of protein alignment and the aligned residues, and the TM-score, which assesses structural similarity based on C-alpha atoms in the aligned residues, yielded different interpretations (Zhang and Skolnick, 2004; Nguyen et al., 2011). Therefore, a high Z-score and low TM-score implies that the alignment is statistically significant, but that the structures do not share the same fold, potentially leading to functional differences. The results indicate that PaWAKL29 in R0.12 differs from its equivalent in West-Indian and Leola™ rootstocks. These structural differences suggest that the protein-OG BAs of these proteins may vary across different rootstocks.

PaWAK4, PaWAK13, PaWAKL10, and PaWAKL29 are candidate defence-related proteins expected to recognise and bind OGs to influence downstream defence pathways, indicated by their negative binding energies in the ligand-binding analysis (**Figure 6D**). Differences were observed between the proteins from different rootstocks, with the Dusa^®^ versions of PaWAK13 and PaWAKL10 predicted to bind OGs more efficiently than the R0.12 counterparts. This finding, combined with transcriptomic analyses, provides multi-omic evidence proposing the involvement of *PaWAK13* and *PaWAKL10* in the successful defence response against *P. cinnamomi*. Additionally, the Leola™ PaWAK13 and PaWAKL10 are predicted to have a higher affinity for OGs than those from Dusa^®^ and R0.12, implying that these proteins may respond more rapidly to pathogen penetration, potentially initiating the defence response earlier.

## 5 Conclusion

This study is the first comprehensive *in silico* characterisation of the complete *PaWAK/WAKL* gene family in avocado, highlighting the involvement of *PaWAK4*, *PaWAK13*, *PaWAKL10, PaWAKL29*, and *PaWAKL31* in the defence response against *P. cinnamomi*. Promoter analyses indicated that the upregulated *PaWAK/WAKLs* are associated with the complex regulatory networks in avocado defence. The structural modelling and binding affinity analyses revealed significant differences among the PaWAK/WAKL proteins across different avocado rootstocks of varying resistance to *P. cinnamomi*. These insights suggest that the continued upregulation of *PaWAK/WAKLs* associated with phytohormone- and stress defence pathways and the efficient recognition of OGs by these proteins may enhance this early defence response in a resistant rootstock. Future research should focus on validating these candidate gene roles through functional assays and exploring their application in a molecular screening tool to aid in the selection of resistant avocado rootstocks for commercial use.

## Data Availability

The original contributions presented in the study are included in the article/**Supplementary Material**. Further inquiries can be directed to the corresponding author/s.

## Supplementary material

Supplementary Figure 1 Scatter plots indicating protein length (amino acids), molecular weight (kD), isoelectric point (pH) and the predicted subcellular localisation of the PaWAK/WAKL proteins. Plas-Plasma membrane; Extr-Extracellular space; Vacu-Vacuole; Chlo-chloroplast; E.R.-Endoplasmic reticulum, Cyto- cytoplasm; Pero-peroxisome.
