# Supplementary figures and images for: In silico characterisation of the avocado WAK/WAKL gene family with a focus on genes involved in defence against Phytophthora cinnamomi
Source: Front Plant Sci. 2025 Jan 29;15:1474781. doi: 10.3389/fpls.2024.1474781 (PMC11814450; doi:10.3389/fpls.2024.1474781)

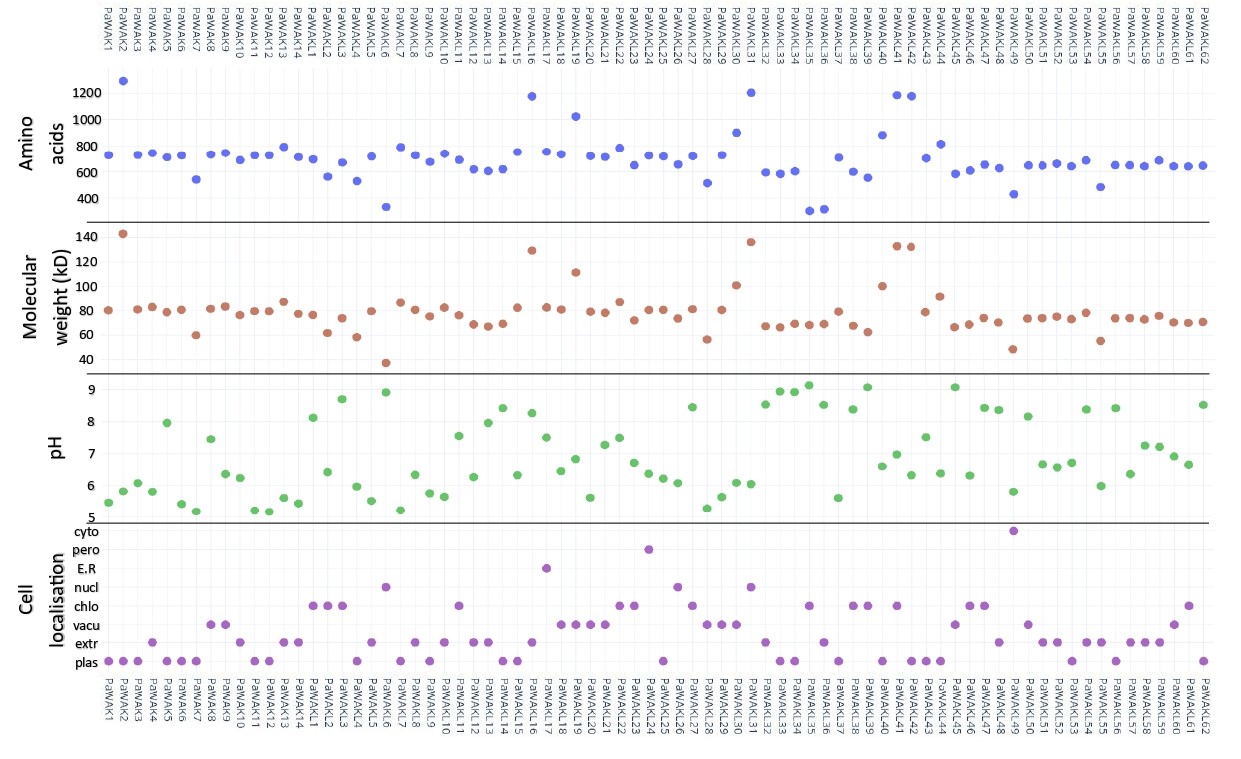

Supplement: Supplementary Figure 1 — Scatter plots indicating protein length (amino acids), molecular weight (kD), isoelectric point (pH) and the predicted subcellular localisation of the PaWAK/WAKL proteins. Plas-Plasma membrane; Extr-Extracellular space; Vacu-Vacuole; Chlo-chloroplast; E.R.-Endoplasmic reticulum, Cyto- cytoplasm; Pero-peroxisome. [file Image1.jpeg]
